# Rapid Identification of Pathogens in Positive Blood Culture of Patients with Sepsis: Review and Meta-Analysis of the Performance of the Sepsityper Kit

**DOI:** 10.1155/2015/827416

**Published:** 2015-04-27

**Authors:** Nils G. Morgenthaler, Markus Kostrzewa

**Affiliations:** ^1^Institut für Experimentelle Endokrinologie, Charité Universitätsmedizin Berlin, Campus Virchow-Klinikum, Augustenburger Platz 1, 13353 Berlin, Germany; ^2^Bruker Daltonik GmbH, Fahrenheitstraße 4, 28359 Bremen, Germany

## Abstract

Sepsis is one of the leading causes of deaths, and rapid identification (ID) of blood stream infection is mandatory to perform adequate antibiotic therapy. The advent of MALDI-TOF Mass Spectrometry for the rapid ID of pathogens was a major breakthrough in microbiology. Recently, this method was combined with extraction methods for pathogens directly from positive blood cultures. This review summarizes the results obtained so far with the commercial Sepsityper sample preparation kit, which is now approved for* in vitro* diagnostic use. Summarizing data from 21 reports, the Sepsityper kit allowed a reliable ID on the species level of 80% of 3320 positive blood culture bottles. Gram negative bacteria resulted consistently in higher ID rates (90%) compared to Gram positive bacteria (76%) or yeast (66%). No relevant misidentifications on the genus level were reported at a log(score)cut-off of 1.6. The Sepsityper kit is a simple and reproducible method which extends the MALDI-TOF technology to positive blood culture specimens and shortens the time to result by several hours or even days. In combination with antibiotic stewardship programs, this rapid ID allows a much faster optimization of antibiotic therapy in patients with sepsis compared to conventional workflows.

## 1. Introduction

Sepsis is one of the leading causes of death in the world with an estimated incidence rate of up to 19 million people worldwide every year [1]. Whilst diagnosis and treatment of sepsis have been improved over the last decades [2], and guidelines are in place to help physicians with this complex syndrome [3], the mortality rate of severe sepsis or septic shock in the intensive care unit (ICU) is still very high (20–50%). Early infection control by the appropriate use of antibiotics is one of the cornerstones of goal directed sepsis therapy, since the survival rate of untreated patients with sepsis decreases by the hour [4]. But the treatment of a patient with antibiotics differs from other medical treatments (e.g., hypertension or diabetes) in a conceptual point of view. Whilst treatment of hypertension is limited to one patient and has no effect on other patients, any antibiotic treatment induces a selection pressure on the bacterium, which may result in a resistant strain that is not confined to the treated patient but will spread to the environment [5]. As more and more pathogens become resistant to many antibiotics, the challenge for the ICU physician is to treat the causative pathogen with the right antibiotic regimen to save the life of his patient and at the same time use antibiotics diligently in an individual patient, as not to jeopardize the future treatment of many other patients [3].

Since information on the pathogen and on antibiotic susceptibility reaches the clinician with a delay of several days, the present practice demands a “blind” initiation of antibiotics, based on the experience of the physician, the probable site of infection, and the resistance patterns known within the respective hospital. To cover as many as possible pathogens, physicians in the ICU often tend to start with broad-spectrum antibiotics, and sometimes with a combination of two or more antibiotics. However, several studies have shown that in most cases combination therapies are not superior to a monotherapy [6–8]. Furthermore, early deescalation of antibiotic therapy is often possible and recommended by existing guidelines [3, 8, 9] and may even reduce mortality in severe sepsis and septic shock [10]. The challenge here is to inform the physician quickly about the kind of pathogen he or she is dealing with, so that the necessary adjustments in therapy can be carried out.

The identification of microorganisms in the microbiology laboratory has fundamentally changed with the routine identification of microorganisms by Matrix-Assisted Laser Desorption Ionization Time-of-Flight Mass Spectrometry (MALDI-TOF MS) [11–13]. Rapid identification (ID) of bacteria [11, 12] and yeast [13] is now possible without time-consuming subculture routines and subsequent phenotypic identification methods, which go partially back to the time of early microbiology pioneers, like Robert Koch. This review outlines the advantage this new technology may bring to the rapid identification of pathogens in positive blood cultures of patients with sepsis.

## 2. Use of Blood Cultures in Sepsis

Blood cultures are an essential part of sepsis management. Existing guidelines give a clear recommendation: “To optimize identification of causative organisms, we recommend obtaining at least two sets of blood cultures (both aerobic and anaerobic bottles) before antimicrobial therapy, with at least one drawn percutaneously and one drawn through each vascular access device, unless the device was recently (<48 h) inserted.” [3] Although only 30–60% of blood cultures in sepsis become positive [14, 15], this method is one with the highest level of evidence in the diagnostic workup of a sepsis patient [3]. The time to positivity of a blood culture varies and depends much on the pathogen load, the type of pathogen, and its growth capacity [16, 17]. Other factors of influence include the volume of cultured blood taken, the presence of polymicrobial infection, the brand of blood culture bottles used, the time it takes for a culture bottle to reach an incubator, and the pretreatment of patients with antibiotics prior to sampling. So the median time to positivity is around 15 hours, but individual bottles may turn positive between just a few hours and several days [16, 17].

After turning positive, most if not all blood culture bottles are processed by Gram staining. Depending on the laboratory, this may be done immediately, several times a day, or only once in the evening. Gram staining takes usually less than 15 minutes, and ideally the results of the Gram staining are communicated to the physician in a timely manner. Sometimes, the information on the type of Gram staining and the form of pathogen allows already antibiotic stewardship to some extent, if such routines are in place in the respective hospital [18].

Next, the positive blood culture is plated out on agar plates, which are often selected also based on the Gram staining of the pathogen. This usually needs overnight culturing, and depending on the organism even longer (e.g., anaerobes). Subsequent identification traditionally is done using biochemical testing. Automated systems, like the Vitek II (Biomerieux, France) or the Phoenix (Becton Dickinson, USA), need at least additional 6–8 hours, but the method is usually performed overnight. Complete identification of rare organisms will often take more than 72 hours for bacteria and more than 60 hours for fungi [16, 17]. The advent of alternative methods like MALDI-TOF MS [19] or molecular identification [17, 20] from agar plates has already greatly reduced time to identification, and MALDI-TOF is now often the reference method in the microbiology laboratory. Here, the MALDI-TOF application directly to positive blood culture fluids offers an even faster identification (12–24 hours) and may therefore directly influence the treatment of patients with sepsis [18, 19, 21, 22].

## 3. Direct Identification of Pathogens from Blood Cultures

Once a blood culture bottle is positive and is ready for Gram staining and further processing for conventional ID, it is now possible to extract sufficient amount of pathogens for direct identification in MALDI-TOF MS without the need of first growing on an agar plate. It is estimated that MALDI-TOF MS requires about 10^5^ of colony forming units (cfu) to have a sufficient amount of bacterial ribosomal proteins to obtain a reliable protein pattern, which is specific for a certain pathogen. After the initial reports [18, 19, 21, 22], there are now several dozens of publications that describe how to extract bacterial protein from positive blood cultures without the contamination of human blood proteins, which would negatively influence the bacterial protein pattern for MALDI-TOF MS. Most of those reports are “home brew” protocols intended for research use only and have not been further validated for clinical routine use.

The regulatory demand on the development process and quality control of* in vitro* diagnostic products increases year by year [23, 24]. As the intended use of these products is often related to the therapeutic management of patients, this poses regulatory limitations on the broader availability of insufficiently validated in-house methods [23, 24]. Traditionally, many research laboratories do not invest too much time and effort in the complex approval process to obtain regulatory clearance. The demand by regulatory bodies to ensure sufficiently high standards for a quality management system is consistently increasing, and particularly the so-called laboratory developed tests may soon be much harder to maintain [23, 24]. Despite higher costs, routine laboratories often use commercial* in vitro* diagnostic products, where regulatory approval is obtained by the manufacturer, who is also responsible to maintain the quality. These products are usually simple to use, have consistent quality, and may allow direct interlaboratory comparison of results, which is not always possible, if a variety of different home brew methods is used.

## 4. The Sepsityper Kit

The commercial Sepsityper kit (Bruker Daltonik GmbH, Bremen, Germany) is a sample preparation method that enables the isolation of bacteria or fungi from a positive blood culture. The method involves the lyses of blood cells, followed by centrifugation and washing steps. The final result is a pellet of bacteria or fungi, which is further processed by standard methods for ID using MALDI-TOF MS [25].

A Medline search for original research papers published until December 2014 in English revealed 21 reports, which have tested the Sepsityper for the extraction of bacteria or yeast (Table 1). Despite the occasional deviation from the instructions for use of the kit, these reports all use the same extraction method, which allows for a direct comparison of the Sepsityper performance. The results of these publications are summarized here.

## 5. Workflow of the Sepsityper Kit

One mL of blood culture fluid is drawn from positive BC bottles and transferred to a 1.5 mL reaction tube (Eppendorf, Hamburg, Germany). After addition of 200 *μ*L of lysis buffer, the sample is vortexed for 10 s followed by a centrifugation for one minute at maximum speed in a centrifuge for PCR reaction vials. The supernatant is discarded and the pellet resuspended in one mL of washing buffer. After a second centrifugation step, the supernatant is discarded and the pellet suspended in 300 *μ*L of distilled water. After addition of 900 *μ*L ethanol to this suspension, the sample is centrifuged for two minutes. The supernatant is discarded, and after repeated centrifugation residual ethanol is carefully removed. Subsequently, the pellet is suspended in 30 *μ*L of 70% formic acid. 30 *μ*L of acetonitrile is added, and the sample is vortext and briefly centrifuged. One microliter of the extract is spotted onto a MALDI target plate and analysed according to the MALDI Biotyper standard procedure according to the manufacturer (Bruker Daltonik GmbH). It is important to mention that the Sepsityper workflow is dependent on the subsequent identification by MALDI-TOF MS and has therefore the same limitations as this MALDI-TOF method with respect to handling and safety of the device.

## 6. Influence of Blood Culture Bottles

The Sepsityper kit was tested and works well with a variety of different blood culture bottles from different commercial manufacturers. Most studies (seventeen in total) used the Bactec system (BD Diagnostics, Sparks, USA) compared to five studies using the BacT/Alert system (Biomerieux, Nürtingen, Germany). Only two reports exist so far on blood bottles from another manufacturer (VersaTREK, TREK Diagnostic Systems, Cleveland) [26, 27]. One author describes difficulties with the BacT/Alert system, particularly with blood culture bottles containing charcoal [28]. BacT/Alert bottles with charcoal are likely to reduce the number of positive IDs with the Sepsityper and should be avoided if rapid ID from blood culture is wanted [28]. This limitation with charcoal containing bottles was also reported by alternative sample preparation methods [29, 30]. It is presently unclear why Bact/Alert bottles with charcoal may not be suitable. The charcoal is carried through the separation steps, and one may speculate that the charcoal absorbs the microorganism and particularly microbial proteins necessary for the ID spectrum of the MALDI-TOF system. This is likely to reduce the pellet needed for a positive ID. Reports on the anaerobic BacT/Alert SN bottles are inconclusive. One study reports difficulties with this type of bottles [31], whilst another report obtained reliable results [32]. However, the studies using the Bactec system tended to report higher identification rates from positive blood cultures compared to those using BacT/Alert bottles, even if those were charcoal-free (see Table 1).

## 7. Influence of Log(score)s Used for Positive ID

All studies used the Biotyper system (Bruker Daltonik GmbH, Bremen, Germany), which comprises the Microflex MALDI-TOF MS hardware in combination with the MALDI Biotyper software bundle and database (Bruker Daltonik GmbH, Bremen, Germany) in several different release versions. One study [33] also compared the performance of the Sepsityper kit in the Vitek MS system (Biomerieux) to the performance in the Biotyper. Both MALDI-TOF MS systems have regulatory approval for IVD use in Europe, and FDA cleared versions exist in the USA. Also many other countries in the world use both methods.

The log(score) cut-off values recommended in these publications by the Biotyper software for a pathogen identification from agar plates are “an acceptable identification to the species level” if the score is ≥2.0 and an “acceptable identification to the genus level” if the score is ≥1.7. Most studies using the Sepsityper for a rapid ID from positive blood cultures deviated to some extent from these log(score)s recommended for agar plates. Many researchers used lower values for correct species ID of 1.7 [34–37], 1.6 [27, 38], or even 1.5 [25, 39] or combined additional criteria with a lower log(score). Some authors required the first two [39] or three pathogen matches listed as results to be identical [32] or to have the species appear several times in the list of possible pathogens [35]; others looked at a log(score) difference of at least 0.3 between the best pathogen match and the next possible candidate [38]. Generally, those lower log(score)s were considered reliable by all investigators and did in very few cases lead to a false positive ID on the species level (see below for details). Since it was not possible to use one log(score) across all published papers, we accepted the final ID of the respective authors as a correct identification. The rationale for our approach is the fact that each laboratory used their own “gold standard” definition for their final ID, and each compared the results obtained by the Sepsityper kit to this gold standard. So the results reported as correct ID by the Sepsityper kit were correct in comparison with MALDI-TOF ID results from isolated colonies on agar plates, or conventional biochemical methods, or 16S rDNA sequencing in case of doubt. As in a real world setting, the Sepsityper kit was always used in combination with the experience of the laboratory, and additional information available (Gram staining). We acknowledge the fact that this may have some influence on the results and consider this a limitation of this review.

Based on these published reports, the recently released MALDI Biotyper software version “MBT compass” and the new software module for the Sepsityper kit have a log(score) of 1.6 as cut-off for positive ID. Furthermore, general improvements to the database, like differentiation of pathogenic bacteria from likely contaminants (e.g.,* Streptococcus pneumoniae* from other* Streptococci* spp. or* Staphylococcus aureus *from other* Staphylococci* spp.), were recently implemented. Further improvements in the identification algorithm of the software for positive blood cultures could include information on the Gram staining to exclude obvious mismatches from the list of suggested pathogens. Two studies reported a considerable increase in correct ID with very low log(score)s using this approach [37, 40] whilst another study used the information on Gram staining to eliminate misidentifications at low scores [27].

## 8. Performance of the Sepsityper

As outlined above, a meta-analysis of the published studies needs to account for different ways of positive pathogen identification, and a direct comparison of the published numbers for a correct ID on the species or genus level of the different studies is a classic situation of “comparing apples with pears.” Furthermore, some reports were inconsistent in their presentation of data, did not always report Gram positive and Gram negative bacteria separately, or included polymicrobial blood culture bottles in their total numbers, whilst others only reported data on monomicrobial blood cultures. A closer look at the raw data, if available, was needed to account for these differences. Therefore numbers and percentages listed in Table 1 may sometimes deviate from those numbers reported in the abstracts or text of the respective paper.

The 21 published studies tested a total of 3320 positive monomicrobial blood culture samples. Of those, 2648 resulted in a reliable ID on the species level (79.8%). Criteria for this were ID data reported based on a log(score) >1.7. If the authors did not report ID data for that cut-off, data at the next higher log(score) reported was taken (e.g., 1.8). In case the authors did only report data for a lower log(score), these numbers were accepted, if the authors report in their study that they felt confident for positive ID on the species level (e.g., see [25, 38, 39]).

Reported data on Gram negative bacteria was available for 18 studies. Out of a total of 1127 positive monomicrobial blood culture bottles with Gram negative bacteria, the Sepsityper method allowed the correct ID of 1010 samples (89.6%).

Data on Gram positive bacteria was available for 17 studies. 2005 positive monomicrobial blood culture bottles contained Gram positive bacteria, of which the Sepsityper method was able to correctly ID 1525 samples (76.1%).

Twelve studies also reported data on fungi. Of those, some had only less than ten cases. Four studies included more than 20 samples [39–42]. From a total of 188 samples the Sepsityper method found 124 positive yeast IDs (65.9%).

The individual studies had a large range of results, starting at 56% positive ID [42] going up to 100% [41]. Both were studies focusing on yeast with relatively small sample size. Some studies report on a very high (≥95%) ID [33, 43, 44]. Other studies report lower percentages of ID, but most are in the range between 70 and 98% (Table 1).

Generally it can be said that the Sepsityper kit performs better in Gram negative than in Gram positive bacteria. In Gram negative bacteria, positive ID rates were generally above 90% and often higher than 95% [27, 31, 33–36, 43–45]. In two studies, Gram positive bacteria were found more reliably than Gram negative bacteria [38, 46]. One study [38] had a rather low number of overall cases, and the results may therefore be biased.

Gram positive bacteria are more difficult to identify with the Sepsityper. The reason why Gram positive bacteria do not always allow a positive ID directly from positive blood cultures is not yet understood. One could speculate that the more robust cell wall decreases the protein extraction efficacy, and slow growth of some species might lead to a very small pellet after the extraction. But even with Gram positive bacteria, some researchers report a successful ID in around 90% or more of positive blood cultures [33, 43, 45].

It seems to be more challenging to obtain reliable ID in yeast. Several approaches were tested to increase the positive rate for yeast. One study introduced two additional washing steps, which seem to eliminate nonyeast protein, resulting in good yeast protein spectra [41]. Another group used twice the amount of protein containing supernatant on the MALDI target. This alone or in combination with additional laser shots on the MALDI-TOF resulted in an increase in correct IDs on top of those IDs already established by the routine procedure [39]. With these modifications, the overall positive ID rate in yeast was about two-thirds of all tested samples.

Whilst under review, another paper using the Sepsityper kit was published by Egli et al., which has similar ID rates for Gram negative (92.6%) but lower ID rates for Gram positive (64%) compared to the results of the meta-analysis of the other studies [47].

## 9. Alternative Protocols for Direct Identification from Positive Blood Cultures

Alternative, laboratory developed methods for extraction from blood culture have been published during the past years. These methods vary in their approach to remove human cellular components and enrich the microbes from blood culture fluid. Saponin [22] or ammonium chloride [48] has been described for lysis of blood cells prior to enrichment of microorganisms from the blood culture fluid. Separation of microorganisms from blood cells can be performed with differential centrifugation and gel separator tubes [19, 49]. Also, simple stepwise sedimentation of blood cells and microorganisms has been described [18, 21, 43, 50]. Some methods used considerably more (5 to 10 mL) blood culture volume to start with [43, 51–54]. Whilst this may increase the chance of obtaining a larger microbial pellet for the MALDI-TOF ID, it also increases the complexity of the method, for instance, the need for more than one centrifuge.

Although most of the alternative approaches have been reported to be successful, none of these methods has been used in the same way in different laboratories, under standardized conditions for sample preparation or data interpretation. The “home brew” methods were applied by the scientists who have developed and optimized them to their conditions. This makes it very difficult to compare and generalize these results; particularly the time to results varies considerably depending on the applied procedures. Schieffer et al. tried a comparison between studies using the Sepsityper kit and those optimizing the sample preparation process individually [27]. This comparison showed that both approaches gave similar results in most bacteria (e.g., 76.7% correct ID for Gram positive cocci using the Sepsityper and 76.3% using a variety of laboratory developed methods). However, the distribution of samples between the two cohorts was skewed, and a single study using 346 yeast specimens with a correct ID of 91.3% had a strong influence on the cohort using laboratory developed methods [53]. So the overall accuracy of this comparison was in favor of the laboratory developed methods (78.2% versus 84.7%). Interestingly, using more blood culture volume to start the extraction procedure was not always leading to a better accuracy [54].

Generally the consumable costs of any laboratory developed tests are most likely lower than that of a commercial product like the Sepsityper; however to look at only the cost of chemicals would be an incomplete assessment of the entire costs related to such a sample extraction method. A significant proportion of overall costs is contributed by the labor expenses of the laboratories. Furthermore, the individual validation of a new method, and the upkeeping of the required quality control, will further contribute to the costs of “home brew” methods. Whilst some laboratories are willing to save some money on the consumable side, others are not willing or able to set up a quality control system for a laboratory developed test following the increasing regulatory requirements as outlined above. From a scientific point of view, both the Sepsityper and the laboratory developed methods give similar results, and the decision to implement one or the other is up to the individual laboratory.

## 10. How Safe Is the Sepsityper?

A potential risk of the product is the misidentification of microorganisms followed by the wrong therapy for the patient. Therefore the safety of the Sepsityper sample preparation kit needs to be assessed in combination with the safety of the Biotyper.

Based on the 21 published reports, the usage of the Sepsityper sample preparation kit does not raise any safety concerns for the intended use of the product. In many studies, there were no misidentifications to the species level compared to the used laboratory standard, which varied in the different publications and was usually either biochemical ID, or MALDI-TOF ID from agar plates, or occasionally 16S rDNA sequencing if needed. In some studies, misidentifications to the species level occurred. A misidentification was initially presumed, if the results obtained by Sepsityper/MALDI-TOF were different from conventional biochemical ID. However, in some cases, ribosomal sequencing of the microorganism indicated that the conventional identification was wrong and the MALDI-TOF identification was correct. In other cases, the microorganisms in question were difficult to identify on the species level by some earlier versions of the Biotyper database. For instance* Salmonella* spp. are generally not identified to the species but only the genus level, and some organisms of the* Streptococcus viridans* group, like* Streptococcus mitis*, were always identified as* Streptococcus pneumonia*, to minimize the risk of missing this more dangerous pathogen in the patient. However, this is a restriction of the Biotyper system and not due to the Sepsityper.

In fact, the robustness of the Sepsityper sample preparation kit in combination with the Biotyper identification method was so strong that most studies lowered the log(score) for a correct identification well below that recommended by the Biotyper software. This reduction increased the efficacy of the product even more, without increasing the safety risk of the method. Log(score)s as low as 1.5 were used in some studies. Based on the published literature evaluation, it seems to be safe and indicated (in terms of increasing the efficacy of the product) to use lower log(score)s than recommended for Biotyper identification from solid media culture. The rationale for this is the fact that the log(score)s of microorganisms obtained directly from positive blood cultures are always reduced compared to the log(score)s obtained from agar plates. The reason for this is the background of blood culture derived peaks (mainly leukocyte proteins) which cannot be totally removed by any method so far. This background of unspecific peaks “dilutes” the specific information and therefore reduces the log(score)s for organisms isolated from blood cultures. The application of lower cut-offs is not a compromise but a logic adoption of the algorithm which has been proven to be valid in a number of publications described in this review. As a result of these studies, a specific blood culture module of the Biotyper software was developed and is now part of the Sepsityper workflow.

## 11. Summary

The Sepsityper sample preparation kit allows the isolation of sufficient microorganisms from positive blood cultures to have enough material for the subsequent generation of protein fingerprinting patterns for MALDI-TOF-based identification. Based on these patterns, the Biotyper software can identify the unknown organism by comparing the pattern to a reference database. This could be shown for bacteria and yeast. In terms of efficacy (percentage of correct identifications compared to established reference methods using subcultured microorganisms from solid phase culture plates), the best performance of the Sepsityper kit could be achieved with Gram negative bacteria. Gram positive bacteria have a consistently lower percentage of correct genus and species identification, but the method nevertheless gives a correct ID in 75% of samples. The correct identification of yeast was possible in two-thirds of the blood cultures tested.

The method is convenient and takes around 30 minutes, depending on the number of samples being processed. The advantage for the user is a faster identification of microorganisms, compared to subculturing of positive blood cultures on solid phase culture plates. The use of the Sepsityper sample preparation kit leads to a reduction in overall time to results from 8 to >48 hours (in some studies >100 hours), depending on the microorganism growth rate on solid phase culture plates. This more rapid time to result is to the advantage of the patient, since, in combination with antibiotic stewardship programs, optimized antibiotic therapy can very often be administered on the basis of a species/genus identification of the underlying microorganisms [55].

## Figures and Tables

**Table 1:** The 21 published reports on the Sepsityper sample preparation kit. BC: blood culture, ID: identification, cfu: colony forming units, LOD: lower limit of detection, and MALDI-TOF MS: Matrix-Assisted Laser Desorption Ionization Time-of-Flight Mass Spectrometry.

Author	Year	Reference	Blood culture system	Gram neg. bacteria *n* (% pos. ID)	Gram pos. bacteria *n* (% pos. ID)	Fungi *n* (% pos. ID)	Total monomicrobial samples *n* (% pos. ID)	Log(score) cut-off used	Comments
Schubert et al.	2011	[25]	Bactec 9240	98 89.8% species	358 86.3% species	17 70.6% species	473 86.5% species	>1.5 species	ID up to 48 hours earlier compared to conventional methods In 18 of 27 mixed cultures at least one isolate was identified correctly Misidentified were 5 *Streptococcus mitis* as *Streptococcus pneumonia *

Kok et al.	2011	[56]	Bactec FX Plus Aerobic/F Anaerobic/F Lytic/10	187 87.2% genus 79.7% species	285 68.4% genus 46.3% species	—	472 75.8% genus 59.5% species	>1.7 genus >2.0 species	All genus results would be reported as species results by present day software Misidentified were 4 *Streptococcus mitis* as *Streptococcus pneumonia, corrected in later databank updates *

Yan et al.	2011	[41]	Bactec FX	—	—	42 100% species	42 100% species	>1.9 species	LOD of yeast detection from positive BC bottles: 5.9 × 10^5^ CFU/ml Two washing steps added prior to the Sepsityper protocol

Buchan et al.	2012	[34]	Bactec Plus Aerobic/F and Bactec Lytic/10	45 97.7% genus 91.0% species	100 80.0% genus 53% species	5 0% genus	150 85.5% genus 64.8% species	>1.7 genus >2.0 species	ID 24–130 h faster as compared to conventional methods Log(score) >1.7 was sufficient for ID on species level, since overall concordance to routine ID was 96.6% to genus and 94.1% to species level Five discrepant results: three *Streptococcus oralis* had *Streptococcus pneumonia* ID by MALDI; two were misidentified by conventional methods (16S sequencing confirmed MALDI)

Juiz et al.	2012	[43]	Bactec	24 95.8% genus 95.8% species	61 96.7% genus 84.7% species	—	85 96.4% genus 87.0% species	>1.7 genus >2.0 species	Sepsityper performed better than an alternative in-house method for sample preparation No correct ID of one *Streptococcus pneumoniae *

Klein et al.	2012	[35]	Bactec 9240 Bactec Plus Aerobic/F, Anaerobic/F, and Peds Plus/F	52 99% species	71 64% species	—	123 82.9% species	>1.7 species (if matching organism stated several times in list)	Sepsityper performed better than alternative in-house method with gel separator tubes ID for Gram negative better than for Gram positive Misidentification of *S. hominis* instead of *S. epidermidis* and *S. hominis* instead of *S. haemolyticus *

Lagacé-Wiens et al.	2012	[36]	BacT/Alert SA BacT/Alert SN	19 100% species	40 80% species	2 50% species	61 85.2% species 88.5% if >1.5	>1.7 species >1.5	Log(score) >1.5 gave 100% concordance to routine ID Mean turnaround time for conventional ID was 40.9 h compared to 6.6 h for MALDI (if positive ID)

Loonen et al.	2012	[31]	BacT/Alert SA BacT/Alert SN	47 95.7% genus 91.5% species	52 65.4% genus 42.3% species	—	99 79.8% genus 65.7% species	>1.7 genus >2.0 species	Sepsityper performed better and was faster than two alternative sample preparation methods. Anaerobe BacT/Alert SN bottles lead to unreliable results, charcoal containing bottles not suitable

Martiny et al.	2012	[38]	Bactec Plus Aerobic Bactec F Lytic Anaerobic	22 72.7% genus 59.1% species	37 81.1% genus 74.3% species	—	59 78.0% genus 68.4% species	>1.4 genus >1.6 species (if 0.3 difference to next in list)	Relatively low number of BC tested. Gram positive bacteria resulted in better ID than Gram negative (unusual report, not confirmed by other studies). In direct comparison an in-house method performed better than Sepsityper. Log(score) cutoffs could be lowered to >1.6 for correct species ID, which increased the correct ID for both methods

Meex et al.	2012	[32]	BacT/Alert SN	40 82.5% species	67 58.2% species	—	107 67.3% species	>1.8 species	Correct species ID accepted at any log(score), if first three database matches were identical No difference between Sepsityper and in-house method using saponin lysis ID of *Salmonella paratyphi B* only to genus level Direct ID faster than conventional method (1–24 h)

Saffert et al.	2012	[37]	Bactec 9240 Bactec Plus Aerobic/F Anaerobic/F Peds Plus/F	35 94% genus 83% species	64 80% genus 70% species	—	99 85% genus 75% species	>1.5 genus >1.7 species	Sepsityper and two in-house methods compared One *Streptococcus viridans* was identified as *Streptococcus pneumoniae *Log(score)s <1.5 in combination with Gram stain results would lead to another 9 correct ID

Chen et al.	2013	[33]	Bactec Plus Aerobic, Bactec F Lytic Anaerobic, and Bactec Myco Lytic	106 99% genus 88.7% species	75 96% genus 72% species	—	181 97.8% genus 81.8% species	>1.6 genus >2.0 species	Sepsityper used with Biotyper generated significantly more accurate identifications than with Vitek MS Only genus level ID in both systems for *Salmonella spec.* In 21 mixed cultures, correct double ID by Biotyper in 5

Haigh et al.	2013	[28]	BacT/Alert 3D FA, BacT/Alert 3D PF, and BacT/Alert SN	123 90% species	168 64% species	6 0%	297 74% species	>1.7 species	Most ID failures resulted from BC bottles with charcoal; therefore BacT/Alert bottles with charcoal are not the optimal culture medium in combination with Sepsityper

Jamal et al.	2013	[46]	Bactec 9240 or BacT/Alert	53 66% species	99 75,8% species	8 50% species	160 75.6% species	>2.0 species	No data on genus identification shown. Definition of positive ID often independent of log(score). The term “misidentified” instead of “unidentified” was used for ID scores <1.5, 10 species not identified by routine method but MALDI

Nonnemann et al.	2013	[40]	Bactec FX	53 88.7% genus 81% species	148 60% genus 44% species	22 77% species	223 64% genus 56% species	>1.6 genus >1.8 species	The fungi experiments contained mainly spiked BC samples A lower cutoff (>1.5 for species ID) was also applied. Combined with Gram staining, a log(score) >1.3 increased Gram positive results to 85% without misidentifications

Tadros and Petrich	2013	[45]	Bactec Aerobic/F, Anaerobic/F, and Peds Plus/F	19 94.7% genus 94.7% species	60 93.3% genus 66.7% species	1 100% genus 100% species	80 93.8% genus 78% species	>1.7 genus >2.0 species	Some Gram positive specimens were excluded from species analysis Total number of samples contained also 7 cerebrospinal fluids spiked into BC bottles and 10 polymicrobial samples

Gorton et al.	2014	[42]	Bactec	—	—	50 76% genus 56% species	50 76% genus 56% species	>1.6 genus >1.8 species	100–200 cfu of yeast was spiked into BC bottles and after positive growth extracted by Sepsityper A lower log(score) (>1.5) for correct species ID and repeated measurements increased ID on species level to 84% Gram staining not sufficient for fungal ID!

Hazelton et al.	2014	[44]	Bactec Plus Aerobic/F, Lytic/10, Anaerobic/F, and Peds Plus/F	64 98.4% species	—	—	64 98.4% species	>2.0 species	Main focus of the study was the use of the Sepsityper kit for direct antibiotic resistance testing in the BD Phoenix system with 97.9% concordance in susceptibility testing

Martinez et al.	2014	[26]	VersaTREK Redox	46 86.9% genus 80.4% species	95 89.4% genus 81.0% species	5 60% genus 60% species	146 87.7% genus 80.1% species	>1.6 genus >1.8 species	Additional washing step added in protocol Anaerobic BC cultures excluded by design Also at least one correct ID in 77% of polymicrobial cultures Misidentification of 4 *Streptococcus viridans* specimens as *S. pneumoniae *

Schieffer et al.	2014	[27]	Bactec Plus Aerobic/F, Standard/10 Aerobic/F, Peds Plus/F, Lytic/10 Anaerobic/F, and VersaTREK Redox	94 97.8% genus 95.7% species	225 95.5% genus 81.7% species	6 50% genus 16% species	325 94.7% genus 84.0% species	>1.6 genus >1.8 species	Sepsityper was used in two hospitals; BC extraction on site; pellet was stored at RT until shipment, MALDI-TOF MS performed off site

Idelevich et al.	2014	[39]	Bactec 9240, Plus Aerobic/F, Plus Anaerobic/F, Mycosis-IC/F, and Peds Plus/F	—	—	24 62.5% species	24 62.5% species	>1.5 species If first two results were identical	Sepsityper protocol was followed without prior washing steps (compare to Yan et al. [41]). Manual shooting and double protein concentration for MALDI were tried out to increase log(score)s >1.7, Mean yeast for positive ID was 4.5 × 10^7^. Results were 23.5 h earlier than standard method Sepsityper pellets generated antibiotic susceptibility profiles in 72.7% of samples 15 h earlier

## References

[1] Adhikari N. K. J., Fowler R. A., Bhagwanjee S., Rubenfeld G. D. (2010). Critical care and the global burden of critical illness in adults. *The Lancet*.

[2] Angus D. C., van der Poll T. (2013). Severe sepsis and septic shock. *The New England Journal of Medicine*.

[3] Dellinger R. P., Levy M. M., Rhodes A. (2013). Surviving sepsis campaign: international guidelines for management of severe sepsis and septic shock, 2012. *Intensive Care Medicine*.

[4] Kumar A., Roberts D., Wood K. E. (2006). Duration of hypotension before initiation of effective antimicrobial therapy is the critical determinant of survival in human septic shock. *Critical Care Medicine*.

[5] Lynch J. P., Clark N. M., Zhanel G. G. (2013). Evolution of antimicrobial resistance among Enterobacteriaceae (focus on extended spectrum *β*-lactamases and carbapenemases). *Expert Opinion on Pharmacotherapy*.

[6] Brunkhorst F. M., Oppert M., Marx G. (2012). Effect of empirical treatment with moxifloxacin and meropenem vs meropenem on sepsis-related organ dysfunction in patients with severe sepsis: a randomized trial. *The Journal of the American Medical Association*.

[7] Paul M., Benuri-Silbiger I., Soares-Weiser K., Leibovici L. (2004). *β* lactam monotherapy versus *β* lactam-aminoglycoside combination therapy for sepsis in immunocompetent patients: systematic review and meta-analysis of randomised trials. *British Medical Journal*.

[8] Harbarth S., Nobre V., Pittet D. (2007). Does antibiotic selection impact patient outcome?. *Clinical Infectious Diseases*.

[9] Heenen S., Jacobs F., Vincent J.-L. (2012). Antibiotic strategies in severe nosocomial sepsis: why do we not de-escalate more often?. *Critical Care Medicine*.

[10] Garnacho-Montero J., Gutiérrez-Pizarraya A., Escoresca-Ortega A. (2014). De-escalation of empirical therapy is associated with lower mortality in patients with severe sepsis and septic shock. *Intensive Care Medicine*.

[11] Seng P., Drancourt M., Gouriet F. (2009). Ongoing revolution in bacteriology: routine identification of bacteria by matrix-assisted laser desorption ionization time-of-flight mass spectrometry. *Clinical Infectious Diseases*.

[12] Mellmann A., Cloud J., Maier T. (2008). Evaluation of matrix-assisted laser desorption ionization-time-of-flight mass spectrometry in comparison to 16S rRNA gene sequencing for species identification of nonfermenting bacteria. *Journal of Clinical Microbiology*.

[13] Marklein G., Josten M., Klanke U. (2009). Matrix-assisted laser desorption ionization-time of flight mass spectrometry for fast and reliable identification of clinical yeast isolates. *Journal of Clinical Microbiology*.

[14] Raad I., Hanna H., Maki D. (2007). Intravascular catheter-related infections: advances in diagnosis, prevention, and management. *Lancet Infectious Diseases*.

[15] Vincent J.-L., Sakr Y., Sprung C. L. (2006). Sepsis in European intensive care units: results of the SOAP study. *Critical Care Medicine*.

[16] Peralta G., Rodríguez-Lera M. J., Garrido J. C., Ansorena L., Roiz M. P. (2006). Time to positivity in blood cultures of adults with *Streptococcus pneumoniae* bacteremia. *BMC Infectious Diseases*.

[17] Afshari A., Schrenzel J., Ieven M., Harbarth S. (2012). Bench-to-bedside review: rapid molecular diagnostics for bloodstream infection—a new frontier?. *Critical Care*.

[18] La Scola B., Raoult D. (2009). Direct identification of bacteria in positive blood culture bottles by matrix-assisted laser desorption ionisation time-of-flight mass spectrometry. *PLoS ONE*.

[19] Moussaoui W., Jaulhac B., Hoffmann A.-M. (2010). Matrix-assisted laser desorption ionization time-of-flight mass spectrometry identifies 90% of bacteria directly from blood culture vials. *Clinical Microbiology and Infection*.

[20] Liesenfeld O., Lehman L., Hunfeld K.-P., Kost G. (2014). Molecular diagnosis of sepsis: new aspects and recent developments. *European Journal of Microbiology and Immunology*.

[21] Christner M., Rohde H., Wolters M., Sobottka I., Wegscheider K., Aepfelbacher M. (2010). Rapid identification of bacteria from positive blood culture bottles by use of matrix-assisted laser desorption-ionization time of flight mass spectrometry fingerprinting. *Journal of Clinical Microbiology*.

[22] Ferroni A., Suarez S., Beretti J.-L. (2010). Real-time identification of bacteria and *Candida* species in positive blood culture broths by matrix-assisted laser desorption ionization-time of flight mass spectrometry. *Journal of Clinical Microbiology*.

[23] FDA (2014). *Draft Guidance for Industry, Food and Drug Administration Staff, and Clinical Laboratories: Framework for Regulatory Oversight of Laboratory Developed Tests (LDTs)*.

[24] European Commission (2012). *Proposal for a Regulation of the European Parliament and of the Council on In Vitro Diagnostic Medical Devices*.

[25] Schubert S., Weinert K., Wagner C. (2011). Novel, improved sample preparation for rapid, direct identification from positive blood cultures using matrix-assisted laser desorption/ionization time-of-flight (MALDI-TOF) mass spectrometry. *The Journal of Molecular Diagnostics*.

[26] Martinez R. M., Bauerle E. R., Fang F. C., Butler-Wu S. M. (2014). Evaluation of three rapid diagnostic methods for direct identification of microorganisms in positive blood cultures. *Journal of Clinical Microbiology*.

[27] Schieffer K., Tan K., Stamper P. (2014). Multicenter evaluation of the Sepsityper extraction kit and MALDI-TOF MS for direct identification of positive blood culture isolates using the BD BACTEC FX and VersaTREK diagnostic blood culture systems. *Journal of Applied Microbiology*.

[28] Haigh J. D., Green I. M., Ball D., Eydmann M., Millar M., Wilks M. (2013). Rapid identification of bacteria from bioMérieux BacT/ALERT blood culture bottles by MALDI-TOF MS. *British Journal of Biomedical Science*.

[29] Szabados F., Michels M., Kaase M., Gatermann S. (2011). The sensitivity of direct identification from positive BacT/ALERT (bioMérieux) blood culture bottles by matrix-assisted laser desorption ionization time-of-flight mass spectrometry is low. *Clinical Microbiology and Infection*.

[30] Schmidt V., Jarosch A., März P., Sander C., Vacata V., Kalka-Moll W. (2012). Rapid identification of bacteria in positive blood culture by matrix-assisted laser desorption ionization time-of-flight mass spectrometry. *European Journal of Clinical Microbiology and Infectious Diseases*.

[31] Loonen A. J. M., Jansz A. R., Stalpers J., Wolffs P. F. G., van den Brule A. J. C. (2012). An evaluation of three processing methods and the effect of reduced culture times for faster direct identification of pathogens from BacT/ALERT blood cultures by MALDI-TOF MS. *European Journal of Clinical Microbiology & Infectious Diseases*.

[32] Meex C., Neuville F., Descy J. (2012). Direct identification of bacteria from BacT/ALERT anaerobic positive blood cultures by MALDI-TOF MS: MALDI Sepsityper kit versus an in-house saponin method for bacterial extraction. *Journal of Medical Microbiology*.

[33] Chen J. H. K., Ho P.-L., Kwan G. S. W. (2013). Direct bacterial identification in positive blood cultures by use of two commercial matrix-assisted laser desorption ionization-time of flight mass spectrometry systems. *Journal of Clinical Microbiology*.

[34] Buchan B. W., Riebe K. M., Ledeboer N. A. (2012). Comparison of the MALDI biotyper system using sepsityper specimen processing to routine microbiological methods for identification of bacteria from positive blood culture bottles. *Journal of Clinical Microbiology*.

[35] Klein S., Zimmermann S., Köhler C., Mischnik A., Alle W., Bode K. A. (2012). Integration of matrix-assisted laser desorption/ionization time-of-flight mass spectrometry in blood culture diagnostics: a fast and effective approach. *Journal of Medical Microbiology*.

[36] Lagacé-Wiens P. R. S., Adam H. J., Karlowsky J. A. (2012). Identification of blood culture isolates directly from positive blood cultures by use of matrix-assisted laser desorption ionization-time of flight mass spectrometry and a commercial extraction system: analysis of performance, cost, and turnaround time. *Journal of Clinical Microbiology*.

[37] Saffert R. T., Cunningham S. A., Mandrekar J., Patel R. (2012). Comparison of three preparatory methods for detection of bacteremia by MALDI-TOF mass spectrometry. *Diagnostic Microbiology and Infectious Disease*.

[38] Martiny D., Dediste A., Vandenberg O. (2012). Comparison of an in-house method and the commercial Sepsityper kit for bacterial identification directly from positive blood culture broths by matrix-assisted laser desorption-ionisation time-of-flight mass spectrometry. *European Journal of Clinical Microbiology & Infectious Diseases*.

[39] Idelevich E. A., Grunewald C. M., Wüllenweber J., Becker K., Coste A. T. (2014). Rapid identification and susceptibility testing of *Candida* spp. from positive blood cultures by combination of direct MALDI-TOF mass spectrometry and direct inoculation of Vitek 2. *PLoS ONE*.

[40] Nonnemann B., Tvede M., Bjarnsholt T. (2013). Identification of pathogenic microorganisms directly from positive blood vials by matrix-assisted laser desorption/ionization time of flight mass spectrometry. *APMIS*.

[41] Yan Y., He Y., Maier T. (2011). Improved identification of yeast species directly from positive blood culture media by combining Sepsityper specimen processing and Microflex analysis with the matrix-assisted laser desorption ionization biotyper system. *Journal of Clinical Microbiology*.

[42] Gorton R. L., Ramnarain P., Barker K. (2014). Comparative analysis of Gram's stain, PNA-FISH and Sepsityper with MALDI-TOF MS for the identification of yeast direct from positive blood cultures. *Mycoses*.

[43] Juiz P. M., Almela M., Melción C. (2012). A comparative study of two different methods of sample preparation for positive blood cultures for the rapid identification of bacteria using MALDI-TOF MS. *European Journal of Clinical Microbiology and Infectious Diseases*.

[44] Hazelton B., Thomas L. C., Olma T. (2014). Rapid and accurate direct antibiotic susceptibility testing of blood culture broths using MALDI Sepsityper combined with the BD Phoenix automated system. *Journal of Medical Microbiology*.

[45] Tadros M., Petrich A. (2013). Evaluation of MALDI-TOF mass spectrometry and Sepsityper Kit for the direct identification of organisms from sterile body fluids in a Canadian pediatric hospital. *Canadian Journal of Infectious Diseases and Medical Microbiology*.

[46] Jamal W., Saleem R., Rotimi V. O. (2013). Rapid identification of pathogens directly from blood culture bottles by Bruker matrix-assisted laser desorption laser ionization-time of flight mass spectrometry versus routine methods. *Diagnostic Microbiology and Infectious Disease*.

[47] Egli A., Osthoff M., Goldenberger D. (2015). Matrix-assisted laser desorption/ionization time-of-flight mass spectrometry (MALDI-TOF) directly from positive blood culture flasks allows rapid identification of bloodstream infections in immunosuppressed hosts. *Transplant Infectious Disease*.

[48] Prod'Hom G., Bizzini A., Durussel C., Bille J., Greub G. (2010). Matrix-assisted laser desorption ionization-time of flight mass spectrometry for direct bacterial identification from positive blood culture pellets. *Journal of Clinical Microbiology*.

[49] Stevenson L. G., Drake S. K., Murray P. R. (2010). Rapid identification of bacteria in positive blood culture broths by matrix-assisted laser desorption ionization-time of flight mass spectrometry. *Journal of Clinical Microbiology*.

[50] Ferreira L., Sánchez-Juanes F., Porras-Guerra I. (2011). Microorganisms direct identification from blood culture by matrix-assisted laser desorption/ionization time-of-flight mass spectrometry. *Clinical Microbiology and Infection*.

[51] March-Rosselló G. A., Muñoz-Moreno M. F., García-Loygorri-Jordán de Urriés M. C., Bratos-Pérez M. A. (2013). A differential centrifugation protocol and validation criterion for enhancing mass spectrometry (MALDI-TOF) results in microbial identification using blood culture growth bottles. *European Journal of Clinical Microbiology and Infectious Diseases*.

[52] Wimmer J. L., Long S. W., Cernoch P. (2012). Strategy for rapid identification and antibiotic susceptibility testing of gram-negative bacteria directly recovered from positive blood cultures using the Bruker MALDI biotyper and the BD phoenix system. *Journal of Clinical Microbiology*.

[53] Spanu T., Posteraro B., Fiori B. (2012). Direct MALDI-TOF mass spectrometry assay of blood culture broths for rapid identification of *Candida* species causing bloodstream infections: an observational study in two large microbiology laboratories. *Journal of Clinical Microbiology*.

[54] Wüppenhorst N., Consoir C., Lörch D., Schneider C. (2012). Direct identification of bacteria from charcoal-containing blood culture bottles using matrix-assisted laser desorption/ionisation time-of-flight mass spectrometry. *European Journal of Clinical Microbiology & Infectious Diseases*.

[55] Perez K. K., Olsen R. J., Musick W. L. (2014). Integrating rapid diagnostics and antimicrobial stewardship improves outcomes in patients with antibiotic-resistant Gram-negative bacteremia. *Journal of Infection*.

[56] Kok J., Thomas L. C., Olma T., Chen S. C. A., Iredell J. R. (2011). Identification of bacteria in blood culture broths using matrix-assisted laser desorption-ionization Sepsityper and time of flight mass spectrometry. *PLoS ONE*.

